# Diabetic retinopathy and diabetic kidney disease, either isolated or associated, impact on the 10-year risk of cardiovascular disease: are we dealing with similar conditions?

**DOI:** 10.20945/2359-4292-2024-0258

**Published:** 2025-05-07

**Authors:** Clara Krummenauer Maraschin, Janine Alessi, Mateus Augusto dos Reis, Gabriela Oliveira Gonçalves Molino, Gabriela Heiden Teló, Beatriz D. Schaan

**Affiliations:** 1 Universidade Federal do Rio Grande do Sul, Porto Alegre, RS, Brasil; 2 Faculdade de Medicina, Pontifícia Universidade Católica do Rio Grande do Sul, Porto Alegre, RS, Brasil; 3 Universidade Feevale, Novo Hamburgo, RS, Brasil; 4 Universidade Federal de Ciências da Saúde de Porto Alegre, Porto Alegre, RS, Brasil; 5 Instituto de Avaliação de Tecnologia em Saúde (IATS) – Conselho Nacional de Desenvolvimento Científico e Tecnológico (CNPq), Brasil; 6 Serviço de Endocrinologia, Hospital de Clínicas de Porto Alegre, Porto Alegre, RS, Brasil

**Keywords:** Diabetes mellitus, Diabetic nephropathies, Diabetic retinopathy, Heart disease risk factors

## Abstract

**Objective:**

To evaluate the association between diabetic retinopathy, diabetic kidney disease, and the 10-year risk of atherosclerotic cardiovascular disease in patients with diabetes.

**Subjects and methods:**

A cross-sectional study was performed involving patients diagnosed with either type 1 or type 2 diabetes mellitus. Participants were classified into four groups: DM (patients without diabetes-related complications), DR (patients with diabetic retinopathy only), DKD (patients with diabetic kidney disease only), and DR + DKD (patients with both diabetic retinopathy and diabetic kidney disease). The primary outcome was the 10-year risk assessment for cardiovascular events, calculated using the American Heart Association’s atherosclerotic cardiovascular disease score.

**Results:**

A total of 571 patients were selected including 128 with type 1 diabetes (average age of 39.4 ± 15.1 years; 48.4% female) and 443 with type 2 diabetes (average age of 59.5 ± 11.9 years; 66.4% female). Among the participants with type 2 diabetes, the cardiovascular risk was 15.2 ± 14.6% for the DM group, 15.7 ± 10.7% for the DR group, 22.5 ± 16.7% for the DKD group, and 21.5 ± 15.1% for the DR + DKD group, reflecting a significantly higher cardiovascular risk in the groups with renal involvement (P <0.001). For those with type 1 diabetes, the DM group had a risk of 6.1 ± 8.9%, the DR group 8.9 ±11.8%, the DKD group 5.4 ± 8.8%, and the DR + DKD group 6.1 ± 9.5%. The mean difference in risk between the groups was not statistically significant.

**Conclusion:**

In patients with type 2 diabetes, those with diabetic kidney disease appeared to have a higher theoretical risk of cardiovascular disease compared to those with only diabetic retinopathy.

## INTRODUCTION

Atherosclerotic cardiovascular disease (ASCVD) is the leading cause of morbidity and mortality worldwide (^1^). Individuals with diabetes mellitus are three to four times more likely to die from cardiovascular disorders than those without the condition (^2^,^3^). Despite significant advances in prevention and treatment, type 2 diabetes continues to be a major risk factor for ASCVD (^4^). People with diabetes utilize twice the healthcare resources compared to those without diabetes, a disparity that is potentially more pronounced in middle-income countries (^5^,^6^). Diabetes-related complications exacerbate societal burdens by increasing both direct medical costs - such as inpatient care, medications, and outpatient visits - and indirect costs, including reduced productivity at work and increased absenteeism (^7^).

The methods for estimating ASCVD risks have evolved over the years (8). Recently updated guidelines for assessing cardiovascular risk recommend the use of a 10-year ASCVD risk calculator, a simplified tool intended to guide treatment recommendations for ASCVD (^9^). However, diabetes-specific factors, like glycated hemoglobin (HbA1c) (^10^), high levels of albuminuria (^11^), and diabetic retinopathy (^12^), have also been identified as predictors of cardiovascular disease (CVD). The estimated prevalence of diabetic retinopathy is approximately 28.5% in the United States and 36.3% in Brazil, while the prevalence of diabetic kidney disease (DKD) in the United States is about 26.2% (^14^,^15^). In light of these facts, there has been an increasing focus on diabetes-related complications and their detrimental effects on cardiovascular health (^16^). These complications have been incorporated into specific cardiovascular risk calculators for both types 1 and 2 diabetes, although the predictive efficiency of these tools varies across different populations (^17^). Recent studies employing clustering based on clinical or genetic biomarkers have identified subtypes of type 2 diabetes that are differentially associated with macro and microvascular complications, potentially aiding in the prediction of patient outcomes (^18^,^19^).

Nonetheless, the relationship between diabetes-related microvascular complications and the 10-year risk of ASCVD remains ambiguous. Therefore, the aim of this study was to evaluate the association between diabetic retinopathy, DKD, and the 10-year risk of ASCVD in individuals with diabetes.

## METHODS

### Design and participants

This ambispective study focused on patients with types 1 and 2 diabetes who were followed at the outpatient Endocrinology clinic of a public hospital in Southern Brazil. Participants, who were involved in regular follow-up, attended the institution to undergo color fundus photography (CFP) for retinopathy screening between 2019 and 2021. Clinical and demographic data were collected during face-to-face interviews with the participants and extracted from electronic medical records at two distinct times (June 2019 and November 2021).

The study included individuals aged 18 years or older who had at least one blood HbA1c measurement, a complete serum laboratory profile for renal function, and whose glycemic control and lipid profile were collected up to a year before the CFP assessment. The criteria excluded patients whose type of diabetes was not clearly established; who had not undergone the requisite laboratory tests prior to the first interview before the CFP; whose CFP results were unclassifiable; and pregnant women. This study adhered to STROBE guidelines (^20^).

### Study procedures

#### Assessment of clinical and demographic aspects

An interview was conducted to gather data on participants’ demographic characteristics (age, sex, race/ethnicity, smoking status, duration of diabetes), concurrent diseases, and medication usage (including antidiabetics, insulin, antihypertensives, lipid-lowering drugs). Patients’ laboratory results were evaluated for creatinine, urea, glucose, HbAlc, total cholesterol, high-density lipoprotein (HDL) cholesterol, triglycerides, and urinary albumin concentration (UAC) levels. Glycated hemoglobin levels were analyzed using the high-performance liquid chromatography method. Data on blood pressure, weight and body mass index (BMI), and CVD were also extracted from electronic medical records, specifically from those appointments in which the patients’ consulting physicians had requested the CFP. The criteria for defining CVD included previous cardiovascular events (coronary heart disease without infarction or heart failure), based on the medical records. In our categorization of CVD, we included only those patients with heart failure or coronary disease who had not experienced a prior myocardial infarction, thus excluding individuals with any significant cardiovascular events. Hypertension was determined by a systolic blood pressure of ≥ 140 mmHg and/or diastolic blood pressure of ≥ 90 mmHg and/or the usage of antihypertensive medication.

#### Assessment of diabetic complications

To evaluate diabetic retinopathy during an in-person appointment, CFPs were captured by a trained technician using a Canon CR-2 camera (Canon Inc., United States). Two images of the posterior segment of each eye - one centered on the macula and the other on the disc (45° field of view) - were obtained after inducing mydriasis with tropicamide 1% eye drops. An ophthalmologist with a specialization in the retina then interpreted the exam. The results were categorized based on the International Council of Ophthalmology Diabetic Retinopathy guidelines. For the analysis, the results of the eye with the most severe findings were considered (^21^).

To assess additional diabetes-related complications, data from medical records were utilized. The presence of DKD was assessed using UAC levels from a random urine sample and estimated glomerular filtration rates (GFR) calculated with the CKD-EPI equation (^22^). A diagnosis of DKD was confirmed for patients with a GFR < 60 mL/min/1.73 m^2^ and/or a UAC value in a urine sample ≥ 14 mg/L (21). Urinary albumin concentration and GFR data were extracted from patients’ medical records, with the samples for the exams collected close to the date of the CFP.

#### Patient stratification

Patients included in the study were divided into four groups based on the presence or absence of diabetes-related complications: DM, patients without diabetes-related complications; DR, patients with only diabetic retinopathy; DKD, patients with only DKD; and DR + DKD, patients with both DKD and diabetic retinopathy.

#### Cardiovascular risk assessment and study outcome

The primary study outcome was the assessment of a 10-year cardiovascular event risk using the ASCVD (Atherosclerotic Cardiovascular Disease) score , proposed by the American Heart Association (AHA). This tool assists healthcare providers in estimating a patient’s 10-year risk of atherosclerotic cardiovascular events, including coronary death, nonfatal myocardial infarction, and fatal or nonfatal stroke, employing the Pooled Cohort Equations. The variables needed for the ASCVD risk calculation include age, sex, race, total cholesterol, HDL cholesterol, blood pressure medication use, diabetes status, and smoking status (^9^). The study used an online calculator for estimating patients’ 10-year cardiovascular risk. A researcher, blinded to patient grouping, entered the data into the calculator, and the results were recorded in a Statistical Package for the Social Sciences (SPSS) spreadsheet. Subsequently, the mean cardiovascular risk for each group was determined, according to the presence or absence of microvascular complications.

### Statistical analysis

The statistical analysis was conducted using SPSS version 20.0 software. The demographics and clinical data that followed a normal distribution were presented as mean ± standard deviation, whereas data with an asymmetric distribution were shown as the median ± interquartile range. The Shapiro-Wilk test was utilized to assess normality. Differences between groups for baseline data and diabetes care quality indicators were assessed using the chi-squared test for categorical variables. Unpaired t-test and Mann-Whitney U-test were used for comparisons of continuous variables.

The study outcomes initially underwent analysis with the non-parametric Kruskal-Wallis test to compare the various groups. Subsequent analysis employed an analysis of covariance to estimate the relationship between different profiles of diabetic complications and the 10-year cardiovascular risk, with the ‘time since diagnosis of diabetes’ as a covariate. The residual normality test found no violations of the normality assumption. The adjusted data are represented as coefficients of linear regression (beta) and their respective 95% confidence intervals (95%CI). An α level of ≤ 0.05 was set to establish statistical significance.

### Ethical aspects

The investigation was conducted following the Helsinki Declaration (2004), adhering to all pertinent guidelines and regulations and received approval from the Research Ethics Committee of *Hospital de Clínicas de Porto Alegre* (no. 2019-0113). All authors entered into a confidentiality agreement regarding data usage, and participants provided informed consent.

## RESULTS

**Figure 1.**
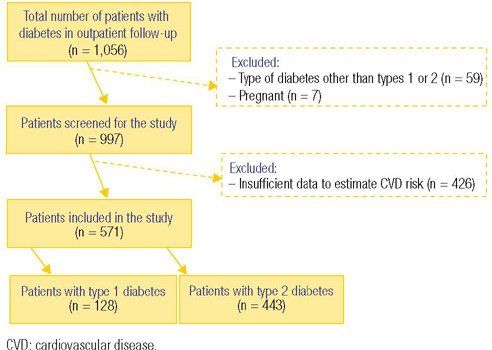
Flow diagram of the study.

The initial cohort comprised 1,056 patients diagnosed with diabetes; however, 426 were were excluded from the final analysis due to insufficient data to estimate their cardiovascular risk (**Figure 1**). Consequently, 571 patients were analyzed, of which 128 had type 1 diabetes (mean age 39.40 ± 15.10 years and 48.4% were female) and 443 had type 2 diabetes (mean age 59.50 ± 11.90 years and 66.40% were female). No differences were observed between the DR, DKD, and DR+DKD groups concerning high blood pressure, smoking status, diabetes duration, and statin usage; hence, these variables were excluded from the linear regression model for adjustment. Additional characteristics are detailed in **Table 1.** The excluded patients did not significantly differ from the included ones in terms of age, sex, and ethnicity (data not shown).

**Table 1.** Baseline characteristics of the study participants

	Total (n = 571)	Type 2 diabetes (n = 443)	Type 1 diabetes (n = 128)
Age (years)	58.00 (46.00-66.00)	60.00 (54.00-68.00)	39.00 (28.00-49.20)
Sex (% female)	356 (62.30)	294 (66.40)	62 (48.40)
Race/ethnicity (% white)	479 (83.90)	369 (83.30)	1 10 (85.90)
BMI (kg/m^2^)	30.00 (25.80-35.10)	31.70 (27.30-36.30)	25.70 (22.50-28.20)
Diabetes duration (years)	14.00 (7.0-21.00)	13.00 (7.00-20.00)	19.00 (12.00-26.80)
HbA1c(%)	8.70 (7.50-9.90)	8.60 (7.30-9.80)	9.10 (7.80-10.30)
Systolic blood pressure (mmHg)	130.00 (120.00-140.00)	130.00 (120.00-144.50)	120.00 (110.00 -130.00)
Diastolic blood pressure (mmHg)	80.00 (70.00-80.00)	80.00 (70.00-85.00)	73.50 (70.00-80.00)
GFR (mL/min/1.73 m^2^)	85.00 (64.00-101.00)	80.00 (59.00-97.00)	99 (80.00-110.00)
Total cholesterol (mg/dL)	176.00 (150.00-208.50)	176.00 (150.00 -207.00)	177.50 (149.50-212.50)
High density lipoprotein cholesterol	45.00 (36.00-55.00)	45.00 (36.00-56.00)	46.00 (38.50-54.00)
Low density lipoprotein cholesterol	102.20 (96.00-111.50)	104.20 (97.10-112.60)	100.30 (95.20-110.50)
Triglycerides (mg/dL)	136.00 (92.00-211.00)	140.00 (95.00-216.50)	122.00 (89.80-195.50)
Smoking	52 (9.10)	41 (9.30)	11 (8.60)
Previous heart failure or coronary disease*	111 (19.70)	103 (23.50)	8 (6.30)
ACE inhibitors use	314 (55.00)	280 (63.20)	34 (26.60)
Antihypertensive use	419 (73.40)	374 (84.40)	45 (35.20)
Statins use	335 (58.70)	298 (67.30)	37 (28.90)
Metformin use		365 (82.40)	-
Insulin use		306 (69.10)	-
iSGLT2 use		47 (10.60)	-

Results are expressed as either the median and interquartile range or n (%).

* Patients with heart failure or coronary disease who have not experienced a previous myocardial infarction are included, thereby excluding individuals with any “hard” cardiovascular events.

BMI: body mass index; HbA1c: hemoglobin A1c; GFR: glomerular filtration rate; ACE: angiotensin-converting enzyme; iSGLT2: inhibitors of the sodium-glucose co-transporter 2.

**Figure 2.**
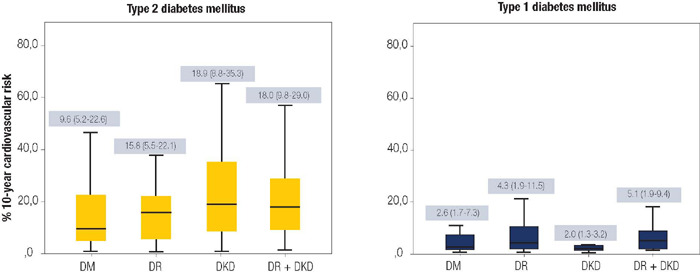
Comparison of the 10-year cardiovascular risks in patients with diabetes, according to the presence or absence of diabetes-related complications. This calculator utilizes the Pooled Cohort Risk Prediction Equations to estimate the risk of atherosclerotic disease.

Among the participants with type 2 diabetes, those in the DM group exhibited a cardiovascular risk of 15.20 ± 14.60%; the DR group, 15.70 ± 10.70%; the DKD group, 22.50 ± 16.70%; and the DR + DKD group, 21.50 ± 15.10%., reflecting a higher cardiovascular risk in the groups with renal involvement (p <0.001). (**Table 2**). *Post hoc* analyses showed that the DKD and DR + DKD groups had a higher 10-year cardiovascular risk compared to the group with no complications and the group with diabetic retinopathyalone. This difference remained significant even after adjusting for age, sex, and the use of angiotensin-converting enzyme (ACE) inhibitors ([R +5.20 [+1.60 to +8.90] and R +4.30 [+0.50 to +8.20] for DKD and DR + DKD groups, respectively) **(Table 3 and Figure 2).**

**Table 2.** Association between diabetes-related microvascular complications and the 10-year risk of atherosclerotic cardiovascular disease in patients with type 2 diabetes

	B (95%CI)	p-value
DM	Reference	
DR	1.56 (−0.99 - +4.11)	0.23
DKD	2.55 (+0.14 - +4.96)	0.04
DR + DKD	−0.75 (−3.93 - +4.45)	0.65
Age (+ 1 year)	0.96 (+0.88 - +1.04)	<0.001
Sex (female)	9.03 (+7.03 - +11.04)	<0.001
ACE inhibitors use	0.70 (−1.23 - +1.04)	0.48

The clinical variables that are integral to the American Heart Association atherosclerotic cardiovascular disease score were not incorporated in the correction model.

The 10-year risk assessment for cardiovascular events was calculated utilizing the American Heart Association atherosclerotic cardiovascular disease score.

95%CI: 95% of confidence interval; ACE: angiotensin-converting enzyme.

DM denotes patients without diabetes-related complications; DR signifies patients with only diabetic retinopathy; DKD represents patients with only diabetic kidney disease; and DR + DKD indicates patients afflicted with both diabetic kidney disease and diabetic retinopathy.

**Table 3.** Association between diabetes-related microvascular complications and the 10-year risk of atherosclerotic cardiovascular disease in patients with type 1 diabetes

	B (95%CI)	P-value
DM	Reference	
DR	−0.44 (−3.62 - + 2.75)	0.78
DKD	0.69 (−2.99 - +4.38)	0.71
DR + DKD	−3.47 (−7.26 - +0.32)	0.07
Age (+ 1 year)	0.39 (+0.30 - +0.48)	<0.001
Sex (female)	5.96 (+3.44 - +8.48)	<0.001
ACE inhibitors use	1.17 (−1.68 - +4.03)	0.42

95%CI: 95% of confidence interval; ACE: angiotensin-converting enzyme.

DM: patients without diabetes-related complications; DR patients with only diabetic retinopathy; DKD: patients with only diabetes-related renal disease; DR + DKD: patients with both diabetic retinopathy and diabetes-related renal disease. ACE: Angiotensin-converting enzyme.

Given the limited sample size of patients with type 1 diabetes included in the study, the analysis should be regarded as exploratory. Among these participants, those in the DM group had a 10-year cardiovascular event risk of 6.10 ± 8.90%, the DR group 8.90 ± 11.80%, the DKD group 5.40 ± 8.80%, and the DR + DKD group 6.10 ± 9.50%. No differences in risk were observed among these groups (**Table 2**).

## DISCUSSION

This study aimed to assess the association between diabetic retinopathy, DKD, and the 10-year risk of ASCVD in patients with diabetes. Given that these chronic complications are linked to poor glycemic control, we hypothesized that individuals with both conditions would demonstrate a higher 10-year estimated risk of ASCVD. Our findings indicate that patients who present with DKD (with or without diabetic retinopathy) exhibit an increased 10-year risk of ASCVD compared to those without diabetes-related complications. Conversely, diabetic retinopathy alone (without DKD) was not associated with a higher estimated cardiovascular risk. This distinction remained after adjusting for potential confounding variables. In individuals with type 1 diabetes, no associations between diabetes-related microvascular complications and the 10-year risk of ASCVD were observed.

The association between DKD and an increased cardiovascular risk, identified in this study, has been corroborated by numerous recent articles . However, the mechanisms underlying this association are not fully understood. In diabetic patients, chronic kidney disease predominantly arises from the pathological changes within the kidney’s vessels, glomeruli, and tubular interstitium (^23^). Hyperglycemia increases the formation of advanced glycation end-products, hypoxia, oxidative damage, activation of the renin-angiotensin-aldosterone system, and the production of inflammatory and fibrotic factors, leading to vascular dysfunction and endothelial damage (^24^,^25^). In the general population, both a decreased GFR and increased albuminuria are recognized as independent risk factors for cardiovascular morbidity and all-cause mortality (^26^,^27^). According to the Chronic Kidney Disease Prognosis Consortium, the likelihood of cardiovascular mortality progressively rises as the estimated GFR decreases: the risk ratios for cardiovascular death escalated from 1.52 in patients with an estimated GFR of 45 to 59 mL/min/1.73m^2^ to 13.51 in those with a GFR of 15 to 29 mL/min/1.73m^2^. The concurrent presence of reduced estimated GFR and albuminuria above 10 mg/g in patients was multiplicatively associated with mortality risk (^28^,^29^).

Approximately 30% to 40% of individuals with diabetes will develop DKD, and nearly half of those with stage 4 or 5 DKD will eventually develop CVD (^30^). These patients often develop risk factors for CVD, including reduced GFR, albuminuria, dyslipidemia, and hypertension, which strongly associates DKD with a heightened 10-year risk of ASCVD.

The association between DKD and cardiovascular risk, although well-established, was underscored by a notable finding in our study: diabetic retinopathy alone does not associated with a heightened 10-year risk of ASCVD. Previous studies suggested that diabetic retinopathy and DKD may reflect equivalent degrees of diabetes progression, which would render diabetic retinopathy a useful marker for predicting DKD (^29^,^30^). Nonetheless, a multicenter case-control study encompassing 4,050 patients at varying stages of renal function decline due to DKD explored the influence of diabetic retinopathy on the deterioration of GFR. This study found that the presence and severity of diabetic retinopathy were risk factors for the onset of DKD (^31^). This suggests that diabetic retinopathy may serve as an early indicator of developing micro-vascular complications in diabetes (^32^), and that the progression of hyperglycemia can lead to vascular dysfunction, thereby worsening kidney function and elevating the risk of CVD.

This study did not uncover any associations between the complications of type 1 diabetes and the 10-year risk of ASCVD. This may be attributed to the study’s limited power, owing to a small sample size, thereby rendering this analysis exploratory. Furthermore, the retrospective nature of the study posed challenges related to potential information bias due to incomplete medical records on physical activity levels. We opted to exclude this variable from our analyses, which may represent a limitation of this study.

## CONCLUSION

This study underscores the necessity of obtaining a deeper understanding of the diverse underlying mechanisms responsible for diabetes-related micro-vascular complications and their association with cardiovascular disease. Such insight is crucial for identifying potential therapeutic targets. In individuals with type 2 diabetes, it was evident that specific microvascular complications impact the 10-year cardiovascular risk differently. Notably, those with renal involvement due to the progression of diabetes are at a theoretically higher risk of cardiovascular disease compared to those with diabetic retinopathy alone. This highlights the critical importance of early diagnosis and treatment of diabetic kidney disease, as well as adhering to recommended glycemic targets, to mitigate health risks and potentially reduce the deterioration of quality of life and life expectancy. Furthermore, to ascertain whether these effects are also prevalent in individuals with type 1 diabetes, a larger cohort is essential. Considering the significant implications of diabetes-related complications and cardiovascular disease, enhancing cardiovascular risk assessment tools emerges as a pivotal clinical need that should inform and guide clinical decision-making.
